# Crystal structure and Hirshfeld surface analysis of 2-phenyl-1*H*-phenanthro[9,10-*d*]imidazol-3-ium benzoate

**DOI:** 10.1107/S2056989020005344

**Published:** 2020-04-24

**Authors:** Ruby Ahmed, Onur Erman Doğan, Farman Ali, Musheer Ahmad, Adeeba Ahmed, Necmi Dege, Irina A. Golenia

**Affiliations:** a Department of Applied Chemistry, ZHCET, Aligarh Muslim University, Aligarh, 202002, UP, India; b Ondokuz Mayıs University, Faculty of Arts and Sciences, Department of Chemistry, 55139 Samsun, Turkey; c Ondokuz Mayıs University, Faculty of Arts and Sciences, Department of Physics, 55139 Samsun, Turkey; d Department of Chemistry, Taras Shecchenko National University of Kyiv, 64, Vladimirska Str., Kiev 01601, Ukraine

**Keywords:** crystal structure, 2-phenyl-1-*H*-phenanthro[9,10-*d*]imidazole, hydrogen bonding, π–π inter­actions

## Abstract

In the title compound, C_21_H_15_N_2_^+^·C_7_H_5_O_2_^−^, 2-phenyl-1*H*-phenanthro[9,10-*d*]imidazole and benzoic acid form an ion pair complex. The system is consolidated by hydrogen bonds along with π–π inter­actions and N—H⋯π inter­actions between the constituent units. For a better understanding of the crystal structure and inter­molecular inter­actions, a Hirshfeld surface analysis was performed.

## Abstract 2

The title compound exists in the crystal as a dimer of ion pairs. Hydrogen bonding and weak π–π inter­actions along with N—H⋯π inter­actions are involved in consolidating this cluster. The three-dimensional crystal structure consists of stepped stacks of dimers of ion pairs associated by C—H⋯π(ring) and slipped π-stacking inter­actions.

## Chemical context   

When phenanthrene is substituted by a heterocyclic moiety, its inter­molecular charge-transfer ability is increased (Xu *et al.*, 2017 ▸). Such a donor–π–acceptor (*D*–π–*A*) arrangement has tunable properties that can be controlled by suitable substituents (Cao *et al.*, 2017 ▸). The presence of a heteroatom such as N, O or S may give electron-rich heterocycles (thio­phene, pyrrole, or furan) or electron-deficient heterocycles (pyridine, phenanthroline) (Xu *et al.*, 2017 ▸). The dipole moment and λ_max_ can be modulated by the selection of *D* and *A*. Thus the photophysical properties can be controlled (Wang *et al.*, 2017 ▸). The inclusion of heterocycles enhances the polarizability, thermal and chemical stabilities of such adducts. The π-conjugated heterocyclic systems increase delocalization, thus enhancing the stability and photophysical properties (Gu *et al.*, 2017 ▸, Zhang *et al.*, 2012 ▸). By proper selection of the heterocyclic substituent, good fluorescence with higher sensitivity can be achieved (Li *et al.*, 2016 ▸; Huang *et al.*, 2012 ▸). The synthesis of selective chromo-fluoro­genic sensors for anions, cations and neutral mol­ecules can be achieved (Chou *et al.*, 2012 ▸; Zhuang *et al.*, 2012 ▸). Herein we report the crystal structure of the title compound, which was synthesized from 2-phenyl-1*H*-phenanthro[9,10-*d*]imidazole and benzoic acid.
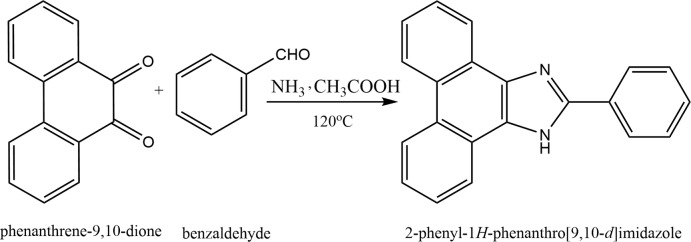


## Structural commentary   

**Figure 1:**
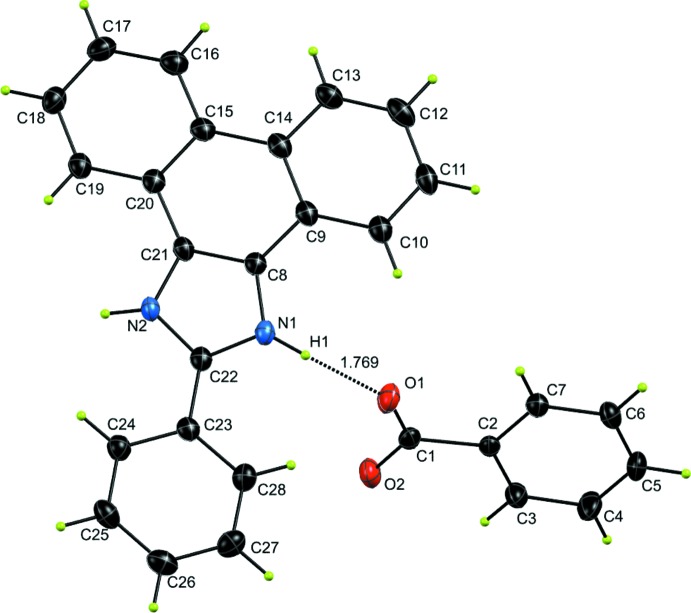
The mol­ecular structure of the title compound with atom labelling. The dashed line indicates the N—H⋯O hydrogen bond. Displacement ellipsoids are drawn at the 50% probability level.

**Figure 2:**
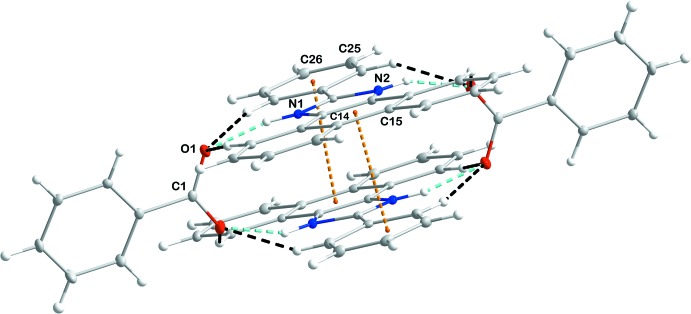
Unit *A* consisting of two entities each of benzoate ions and M1 moieties, linked by hydrogen bonds and π–π inter­actions.

The structure of the title compound is shown in Fig. 1 ▸. The proton from benzoic acid (BA) is completely transferred to the N atom of the imidazole ring of 2-phenyl-1-*H*-phenanthro[9,10-*d*]imidazole (M1), leading to the formation of a M1^+^BA^−^ co-crystal. The space group is monoclinic, *P*2_1_/*n* and two asymmetric units, two M1^+^ ions and two benzoate ions, are combined in an inversion dimer of ion pairs (unit *A*, Fig. 2 ▸). The benzoate ion and M1^+^ are nearly perpendicular [67.82 (4)°] to one another and the torsional angle C1—O1—N1—C22 is 78.24 **(su?)**°. Unit *A* is stabilized by hydrogen bonds (N1—H1⋯O1, 1.77 Å, and N2—H2⋯O2, 1.83 Å; Fig. 2 ▸). Beside the hydrogen bonds, there are weak π inter­actions between the two M^+^1 moieties [inter­centroid separations between the C23–C28 and C8/C9/C14/C15/C20/C21 rings = 3.4590 (9) Å].

## Supra­molecular features   

**Figure 3:**
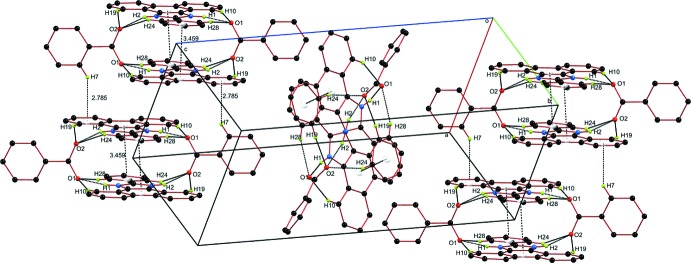
Supra­molecular structure showing *A* units stacked over adjacent rows of *A* units running perpendicular to each other.

In the crystal, the *A* units are associated through weak, slipped, π-stacking inter­actions between the C9–C14 benzene rings and N1/C22/N2/C21/C8 imidazole rings across inversion centers [centroid–centroid distance = 3.5675 (9) Å, dihedral angle = 1.57 (8)°, slippage = 1.532 Å). The stepped stacks thus formed extend alternately in the directions of the normals to (111) and (1
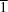
1) and are connected *via* C7—H7⋯*Cg*4 inter­actions (Table 1 ▸, Fig. 3 ▸).

## Hirshfeld surface analysis   

**Figure 4:**
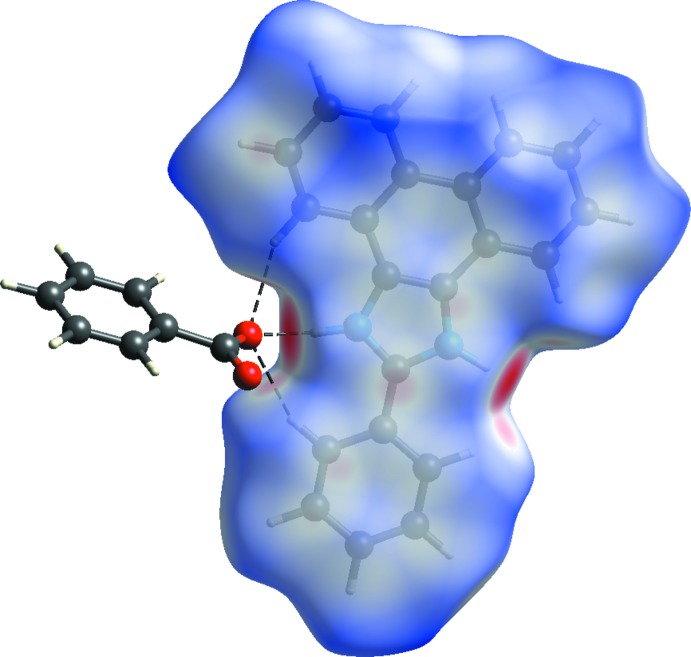
View of the three-dimensional Hirshfeld surface of the title compound plotted over *d*_norm_.

**Figure 5:**
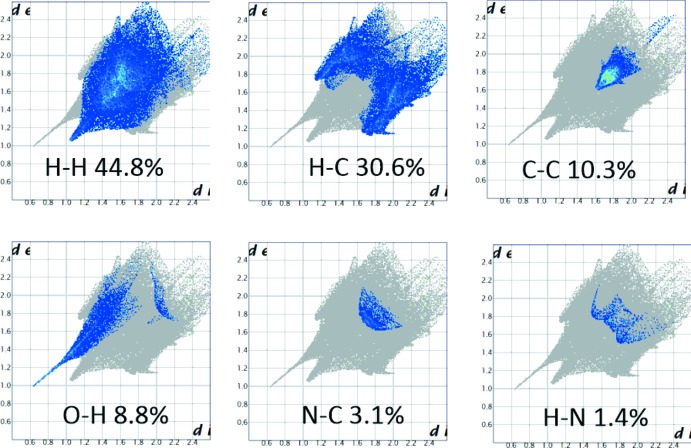
Two-dimensional fingerprint plots of the crystal with the relative contributions of the atom pairs to the Hirshfeld surface.

The Hirshfeld surfaces provide an extended qualitative and qu­anti­tative analysis of the inter­actions between the constituents of the co-crystal. The analysis shows the presence of C—H⋯O and N—H⋯O hydrogen bonds leading to multidirectional inter­actions to form the three-dimensional structure. The red spots in the Hirshfeld surface (Fig. 4 ▸) are centered on the N1—H1⋯O1, C10—H10⋯O1 and C28—H28⋯O1 inter­actions of the benzoate ion with the phenanthrene and with the N—H of the imidazole. Their bond lengths are 1.77, 2.40, and 2.48 Å, respectively. The fingerprint plots (Fig. 5 ▸) show the percentage contribution of the various inter­actions. Those of H⋯H and H⋯C dominate at 44.8% and 30.6%, respectively. The H⋯O inter­actions involve oxygen atoms from the benzoate anion and the N—H group of the imidazole ring of M1^+^.

## Database survey   

A search of the Cambridge Structural database (CSD, version 5.41, update November 2019; Groom *et al.*, 2016 ▸) for the 2,3-di­hydro-1*H*-phenanthro[9,10-*d*]imidazole moiety revealed 45 hits of which the most similar to the title compound are imidazole derivatives (CEZWEL: Mormul *et al.*, 2013 ▸; ODEDAD: Li *et al.*, 2016 ▸; QORJUD: Tapu *et al.*, 2009 ▸; REKXOX: Akula *et al.*, 2017 ▸; YUMTEG: Ullah *et al.*, 2009 ▸; ZACSAA: Therrien *et al.*, 2014 ▸). The N—C bond lengths of the imidazole ring in these structures vary from 1.312 (2) to 1.365 (2) Å. The mol­ecular conformations of these structures are also planar.

## Synthesis and crystallization   

A condensation reaction was performed between equimolar qu­anti­ties of phenanthrene-9,10-dione and benzaldehyde. 1 mmol of phenanthrene-9,10-dione, 1 mmol of benzaldehyde, 5 mmol of ammonium acetate and 30 mL of glacial acetic acid were added to single-neck 100 mL round-bottom flask. The mixture was refluxed for 12 h under nitro­gen. After completion of the reaction, the reaction mixture was cooled to room temperature and then 50 mL of deionized cold water were added. The product precipitated out as pale-brown solid. The solid product was filtered, washed with deionized water and dried in a vacuum oven to give 2-phenyl-1*H*-phenanthro[9,10-*d*]imidazole (M1) as the final product. Crystals were prepared using 20 mg of M1 and 20 mg of benzoic acid dissolved in 5mL of ethanol. The clear solution was left undisturbed for crystallization. Fine crystals were obtained after 15 days.

## Refinement   

Crystal data, data collection and structure refinement details are summarized in Table 2 ▸. The NH hydrogen atoms were located in difference-Fourier maps and, together with the carbon-bound hydrogen atoms, were included as riding contributions in calculated positions [N—H = 0.86, C—H = 0.93 Å; *U*_iso_(H) = 1.2*U*_eq_(C,N)].

## Supplementary Material

Crystal structure: contains datablock(s) I, global. DOI: 10.1107/S2056989020005344/mw2157sup1.cif

Structure factors: contains datablock(s) I. DOI: 10.1107/S2056989020005344/mw2157Isup2.hkl

CCDC reference: 1997348

Additional supporting information:  crystallographic information; 3D view; checkCIF report

## Figures and Tables

**Table 1:** Hydrogen-bond geometry (Å, °) *Cg*4 is the centroid of the C15–C20 benzene ring.

*D*—H⋯*A*	*D*—H	H⋯*A*	*D*⋯*A*	*D*—H⋯*A*
N1—H1⋯O1	0.86	1.77	2.6159 (17)	168
N2—H2⋯O2^i^	0.86	1.83	2.6523 (16)	158
C7—H7⋯*Cg*4^ii^	0.93	2.79	3.585 (2)	145
C10—H10⋯O1	0.93	2.40	3.265 (2)	155
C19—H19⋯O2^i^	0.93	2.54	3.372 (2)	150
C24—H24⋯O2^i^	0.93	2.50	3.343 (2)	152
C28—H28⋯O1	0.93	2.48	3.365 (2)	159

Symmetry codes: (i) 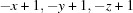; (ii) 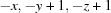.

**Table 2:** Experimental details

Crystal data	
Chemical formula	C_21_H_15_N_2_^+^·C_7_H_5_O_2_^−^
*M*_r_	416.46
Crystal system, space group	Monoclinic, *P*2_1_/*n*
Temperature (K)	100
*a*, *b*, *c* (Å)	9.4693 (4), 8.7384 (3), 24.5049 (9)
β (°)	91.792 (1)
*V* (Å^3^)	2026.70 (13)
*Z*	4
Radiation type	Mo *K*α
μ (mm^−1^)	0.09
Crystal size (mm)	0.39 × 0.28 × 0.17

Data collection	
Diffractometer	Bruker APEXII CCD
Absorption correction	Multi-scan (*SADABS*; Bruker, 2016 ▸)
*T*_min_, *T*_max_	0.708, 0.746
No. of measured, independent and observed [*I* > 2σ(*I*)] reflections	25446, 3979, 3269
*R*_int_	0.046
(sin θ/λ)_max_ (Å^−1^)	0.617

Refinement	
*R*[*F* ^2^ > 2σ(*F* ^2^)], *wR*(*F* ^2^), *S*	0.041, 0.104, 1.10
No. of reflections	3979
No. of parameters	289
H-atom treatment	H-atom parameters constrained
Δρ_max_, Δρ_min_ (e Å^−3^)	0.23, −0.33

Computer programs: *APEX2* and *SAINT* (Bruker, 2016 ▸), *SHELXT2018/3* (Sheldrick, 2015*a* ▸), *SHELXL2018/3* (Sheldrick, 2015*b* ▸), *Mercury* (Macrae *et al.*, 2020 ▸), *WinGX* (Farrugia, 2012 ▸) and *PLATON* (Spek, 2020 ▸).

## supplementary crystallographic information

### Crystal data

**Table:**

C_21_H_15_N_2_^+^·C_7_H_5_O_2_^−^	*F*(000) = 872
*M**_r_* = 416.46	*D*_x_ = 1.365 Mg m^−^^3^
Monoclinic, *P*2_1_/*n*	Mo *K*α radiation, λ = 0.71073 Å
*a* = 9.4693 (4) Å	Cell parameters from 9121 reflections
*b* = 8.7384 (3) Å	θ = 3.2–28.1°
*c* = 24.5049 (9) Å	µ = 0.09 mm^−^^1^
β = 91.792 (1)°	*T* = 100 K
*V* = 2026.70 (13) Å^3^	Block, pink
*Z* = 4	0.39 × 0.28 × 0.17 mm

### Data collection

**Table:**

Bruker APEXII CCD diffractometer	3269 reflections with *I* > 2σ(*I*)
φ and ω scans	*R*_int_ = 0.046
Absorption correction: multi-scan (SADABS; Bruker, 2016)	θ_max_ = 26.0°, θ_min_ = 2.3°
*T*_min_ = 0.708, *T*_max_ = 0.746	*h* = −11→11
25446 measured reflections	*k* = −10→10
3979 independent reflections	*l* = −30→30

### Refinement

**Table:**

Refinement on *F*^2^	Primary atom site location: iterative
Least-squares matrix: full	Hydrogen site location: inferred from neighbouring sites
*R*[*F*^2^ > 2σ(*F*^2^)] = 0.041	H-atom parameters constrained
*wR*(*F*^2^) = 0.104	*w* = 1/[σ^2^(*F*_o_^2^) + (0.0356*P*)^2^ + 1.042*P*] where *P* = (*F*_o_^2^ + 2*F*_c_^2^)/3
*S* = 1.10	(Δ/σ)_max_ < 0.001
3979 reflections	Δρ_max_ = 0.23 e Å^−^^3^
289 parameters	Δρ_min_ = −0.33 e Å^−^^3^
0 restraints	

### Special details

**Table:**

Geometry. All esds (except the esd in the dihedral angle between two l.s. planes) are estimated using the full covariance matrix. The cell esds are taken into account individually in the estimation of esds in distances, angles and torsion angles; correlations between esds in cell parameters are only used when they are defined by crystal symmetry. An approximate (isotropic) treatment of cell esds is used for estimating esds involving l.s. planes.

### Fractional atomic coordinates and isotropic or equivalent isotropic displacement parameters (Å^2^)

**Table:**

	*x*	*y*	*z*	*U*_iso_*/*U*_eq_	
O1	0.23573 (12)	0.35667 (14)	0.39271 (5)	0.0272 (3)	
O2	0.43116 (12)	0.21182 (14)	0.39047 (5)	0.0277 (3)	
N1	0.27589 (13)	0.51384 (15)	0.48272 (5)	0.0174 (3)	
H1	0.259534	0.474240	0.451017	0.021*	
N2	0.36868 (13)	0.65362 (15)	0.54849 (5)	0.0177 (3)	
H2	0.421794	0.718335	0.565817	0.021*	
C1	0.30537 (16)	0.24344 (19)	0.37667 (6)	0.0190 (3)	
C2	0.23159 (16)	0.13699 (18)	0.33666 (6)	0.0174 (3)	
C3	0.30647 (17)	0.0201 (2)	0.31269 (7)	0.0260 (4)	
H3	0.400888	0.004987	0.322848	0.031*	
C4	0.24293 (19)	−0.0746 (2)	0.27379 (7)	0.0327 (4)	
H4	0.294737	−0.151762	0.257587	0.039*	
C5	0.10194 (18)	−0.0537 (2)	0.25920 (7)	0.0261 (4)	
H5	0.059013	−0.115598	0.232633	0.031*	
C6	0.02506 (17)	0.05930 (19)	0.28422 (7)	0.0225 (4)	
H6	−0.070461	0.071109	0.275216	0.027*	
C7	0.08921 (16)	0.15524 (18)	0.32262 (6)	0.0194 (3)	
H7	0.037017	0.231785	0.338992	0.023*	
C8	0.20749 (15)	0.47649 (18)	0.52979 (6)	0.0172 (3)	
C9	0.09665 (15)	0.36873 (18)	0.53830 (6)	0.0185 (3)	
C10	0.03891 (16)	0.27684 (18)	0.49600 (7)	0.0214 (4)	
H10	0.070829	0.286745	0.460668	0.026*	
C11	−0.06499 (16)	0.17229 (19)	0.50718 (7)	0.0241 (4)	
H11	−0.102982	0.111124	0.479346	0.029*	
C12	−0.11351 (17)	0.1577 (2)	0.56009 (7)	0.0267 (4)	
H12	−0.183127	0.086184	0.567421	0.032*	
C13	−0.05897 (17)	0.2486 (2)	0.60154 (7)	0.0243 (4)	
H13	−0.093418	0.238283	0.636479	0.029*	
C14	0.04783 (15)	0.35686 (18)	0.59221 (7)	0.0201 (3)	
C15	0.10846 (15)	0.45281 (18)	0.63613 (6)	0.0197 (3)	
C16	0.06231 (17)	0.4443 (2)	0.69008 (7)	0.0244 (4)	
H16	−0.010480	0.377451	0.698225	0.029*	
C17	0.12253 (17)	0.5328 (2)	0.73112 (7)	0.0263 (4)	
H17	0.090928	0.523517	0.766521	0.032*	
C18	0.23025 (17)	0.6361 (2)	0.72023 (7)	0.0243 (4)	
H18	0.269913	0.695635	0.748191	0.029*	
C19	0.27759 (16)	0.64963 (19)	0.66800 (6)	0.0213 (4)	
H19	0.348675	0.719290	0.660521	0.026*	
C20	0.21874 (15)	0.55833 (18)	0.62580 (6)	0.0187 (3)	
C21	0.26552 (15)	0.56429 (18)	0.57097 (6)	0.0173 (3)	
C22	0.37202 (15)	0.62210 (18)	0.49467 (6)	0.0174 (3)	
C23	0.46252 (15)	0.69837 (18)	0.45586 (6)	0.0180 (3)	
C24	0.55209 (16)	0.81660 (19)	0.47343 (7)	0.0224 (4)	
H24	0.554159	0.846890	0.509828	0.027*	
C25	0.63769 (17)	0.8886 (2)	0.43664 (7)	0.0264 (4)	
H25	0.697088	0.967356	0.448523	0.032*	
C26	0.63607 (17)	0.8448 (2)	0.38243 (7)	0.0264 (4)	
H26	0.695022	0.892778	0.358048	0.032*	
C27	0.54566 (18)	0.7287 (2)	0.36468 (7)	0.0260 (4)	
H27	0.543584	0.699591	0.328159	0.031*	
C28	0.45880 (17)	0.65616 (19)	0.40079 (7)	0.0224 (4)	
H28	0.397852	0.579232	0.388489	0.027*	

### Atomic displacement parameters (Å^2^)

**Table:**

	*U*^11^	*U*^22^	*U*^33^	*U*^12^	*U*^13^	*U*^23^
O1	0.0306 (6)	0.0247 (6)	0.0259 (6)	−0.0004 (5)	−0.0033 (5)	−0.0090 (5)
O2	0.0214 (6)	0.0332 (7)	0.0279 (6)	−0.0035 (5)	−0.0069 (5)	−0.0051 (5)
N1	0.0161 (6)	0.0172 (7)	0.0185 (7)	0.0026 (5)	−0.0037 (5)	−0.0023 (5)
N2	0.0151 (6)	0.0177 (7)	0.0200 (7)	−0.0006 (5)	−0.0035 (5)	−0.0032 (5)
C1	0.0211 (8)	0.0216 (8)	0.0144 (7)	−0.0032 (7)	0.0002 (6)	0.0011 (6)
C2	0.0184 (7)	0.0192 (8)	0.0146 (7)	−0.0039 (6)	0.0010 (6)	0.0011 (6)
C3	0.0166 (7)	0.0336 (10)	0.0277 (9)	0.0000 (7)	−0.0019 (7)	−0.0089 (8)
C4	0.0264 (9)	0.0371 (11)	0.0343 (10)	0.0034 (8)	−0.0006 (8)	−0.0185 (9)
C5	0.0269 (9)	0.0285 (9)	0.0226 (8)	−0.0054 (7)	−0.0046 (7)	−0.0076 (7)
C6	0.0180 (7)	0.0245 (9)	0.0245 (8)	−0.0022 (7)	−0.0051 (7)	0.0006 (7)
C7	0.0192 (8)	0.0187 (8)	0.0203 (8)	0.0010 (6)	−0.0007 (6)	−0.0003 (6)
C8	0.0145 (7)	0.0173 (8)	0.0197 (8)	0.0042 (6)	−0.0019 (6)	−0.0001 (6)
C9	0.0143 (7)	0.0156 (8)	0.0254 (8)	0.0045 (6)	−0.0036 (6)	0.0004 (6)
C10	0.0177 (8)	0.0183 (8)	0.0281 (9)	0.0042 (6)	−0.0035 (7)	−0.0017 (7)
C11	0.0182 (8)	0.0171 (8)	0.0364 (10)	0.0028 (7)	−0.0075 (7)	−0.0035 (7)
C12	0.0178 (8)	0.0200 (9)	0.0420 (11)	0.0001 (7)	−0.0019 (7)	0.0058 (8)
C13	0.0185 (8)	0.0255 (9)	0.0288 (9)	0.0015 (7)	−0.0009 (7)	0.0054 (7)
C14	0.0141 (7)	0.0181 (8)	0.0278 (9)	0.0046 (6)	−0.0025 (6)	0.0019 (7)
C15	0.0147 (7)	0.0207 (8)	0.0234 (8)	0.0060 (6)	−0.0007 (6)	0.0013 (7)
C16	0.0192 (8)	0.0275 (9)	0.0264 (9)	0.0037 (7)	0.0013 (7)	0.0027 (7)
C17	0.0241 (8)	0.0347 (10)	0.0201 (8)	0.0076 (8)	0.0022 (7)	0.0002 (7)
C18	0.0217 (8)	0.0299 (9)	0.0212 (8)	0.0068 (7)	−0.0044 (7)	−0.0032 (7)
C19	0.0162 (7)	0.0227 (9)	0.0247 (9)	0.0040 (6)	−0.0035 (6)	−0.0023 (7)
C20	0.0152 (7)	0.0190 (8)	0.0217 (8)	0.0064 (6)	−0.0033 (6)	−0.0002 (6)
C21	0.0137 (7)	0.0166 (8)	0.0214 (8)	0.0026 (6)	−0.0032 (6)	0.0000 (6)
C22	0.0150 (7)	0.0157 (8)	0.0214 (8)	0.0051 (6)	−0.0032 (6)	−0.0016 (6)
C23	0.0144 (7)	0.0171 (8)	0.0224 (8)	0.0051 (6)	−0.0021 (6)	0.0009 (6)
C24	0.0190 (8)	0.0243 (9)	0.0236 (8)	0.0014 (7)	−0.0024 (7)	−0.0011 (7)
C25	0.0194 (8)	0.0265 (9)	0.0330 (10)	−0.0021 (7)	−0.0045 (7)	0.0038 (8)
C26	0.0203 (8)	0.0286 (10)	0.0304 (9)	0.0034 (7)	0.0014 (7)	0.0097 (8)
C27	0.0301 (9)	0.0264 (9)	0.0215 (8)	0.0057 (7)	0.0000 (7)	0.0018 (7)
C28	0.0234 (8)	0.0191 (8)	0.0243 (8)	0.0021 (7)	−0.0029 (7)	−0.0011 (7)

### Geometric parameters (Å, º)

**Table:**

O1—C1	1.2588 (19)	C12—C13	1.377 (2)
O2—C1	1.2587 (19)	C12—H12	0.9300
N1—C22	1.339 (2)	C13—C14	1.409 (2)
N1—C8	1.380 (2)	C13—H13	0.9300
N1—H1	0.8600	C14—C15	1.467 (2)
N2—C22	1.349 (2)	C15—C16	1.407 (2)
N2—C21	1.379 (2)	C15—C20	1.422 (2)
N2—H2	0.8600	C16—C17	1.378 (2)
C1—C2	1.507 (2)	C16—H16	0.9300
C2—C3	1.384 (2)	C17—C18	1.394 (2)
C2—C7	1.390 (2)	C17—H17	0.9300
C3—C4	1.386 (2)	C18—C19	1.374 (2)
C3—H3	0.9300	C18—H18	0.9300
C4—C5	1.383 (2)	C19—C20	1.407 (2)
C4—H4	0.9300	C19—H19	0.9300
C5—C6	1.382 (2)	C20—C21	1.429 (2)
C5—H5	0.9300	C22—C23	1.461 (2)
C6—C7	1.386 (2)	C23—C24	1.396 (2)
C6—H6	0.9300	C23—C28	1.398 (2)
C7—H7	0.9300	C24—C25	1.382 (2)
C8—C21	1.369 (2)	C24—H24	0.9300
C8—C9	1.430 (2)	C25—C26	1.382 (2)
C9—C10	1.408 (2)	C25—H25	0.9300
C9—C14	1.417 (2)	C26—C27	1.389 (2)
C10—C11	1.376 (2)	C26—H26	0.9300
C10—H10	0.9300	C27—C28	1.381 (2)
C11—C12	1.395 (2)	C27—H27	0.9300
C11—H11	0.9300	C28—H28	0.9300
			
C22—N1—C8	108.53 (13)	C13—C14—C9	117.21 (15)
C22—N1—H1	125.7	C13—C14—C15	122.08 (15)
C8—N1—H1	125.7	C9—C14—C15	120.70 (14)
C22—N2—C21	108.28 (13)	C16—C15—C20	116.92 (15)
C22—N2—H2	125.9	C16—C15—C14	122.22 (15)
C21—N2—H2	125.9	C20—C15—C14	120.86 (14)
O2—C1—O1	126.11 (15)	C17—C16—C15	121.50 (16)
O2—C1—C2	117.05 (14)	C17—C16—H16	119.3
O1—C1—C2	116.84 (14)	C15—C16—H16	119.3
C3—C2—C7	119.04 (14)	C16—C17—C18	120.82 (16)
C3—C2—C1	119.87 (14)	C16—C17—H17	119.6
C7—C2—C1	121.09 (14)	C18—C17—H17	119.6
C2—C3—C4	121.02 (15)	C19—C18—C17	119.72 (16)
C2—C3—H3	119.5	C19—C18—H18	120.1
C4—C3—H3	119.5	C17—C18—H18	120.1
C5—C4—C3	119.58 (16)	C18—C19—C20	120.15 (16)
C5—C4—H4	120.2	C18—C19—H19	119.9
C3—C4—H4	120.2	C20—C19—H19	119.9
C6—C5—C4	119.83 (15)	C19—C20—C15	120.88 (15)
C6—C5—H5	120.1	C19—C20—C21	122.89 (15)
C4—C5—H5	120.1	C15—C20—C21	116.23 (14)
C5—C6—C7	120.51 (15)	C8—C21—N2	107.21 (14)
C5—C6—H6	119.7	C8—C21—C20	122.93 (14)
C7—C6—H6	119.7	N2—C21—C20	129.85 (14)
C6—C7—C2	119.97 (15)	N1—C22—N2	108.77 (14)
C6—C7—H7	120.0	N1—C22—C23	126.05 (14)
C2—C7—H7	120.0	N2—C22—C23	125.15 (14)
C21—C8—N1	107.20 (13)	C24—C23—C28	119.31 (15)
C21—C8—C9	122.75 (15)	C24—C23—C22	119.99 (14)
N1—C8—C9	130.06 (14)	C28—C23—C22	120.69 (14)
C10—C9—C14	120.94 (15)	C25—C24—C23	119.90 (16)
C10—C9—C8	122.55 (15)	C25—C24—H24	120.0
C14—C9—C8	116.51 (14)	C23—C24—H24	120.0
C11—C10—C9	119.71 (16)	C26—C25—C24	120.80 (16)
C11—C10—H10	120.1	C26—C25—H25	119.6
C9—C10—H10	120.1	C24—C25—H25	119.6
C10—C11—C12	120.25 (16)	C25—C26—C27	119.43 (16)
C10—C11—H11	119.9	C25—C26—H26	120.3
C12—C11—H11	119.9	C27—C26—H26	120.3
C13—C12—C11	120.41 (16)	C28—C27—C26	120.56 (16)
C13—C12—H12	119.8	C28—C27—H27	119.7
C11—C12—H12	119.8	C26—C27—H27	119.7
C12—C13—C14	121.48 (16)	C27—C28—C23	119.98 (16)
C12—C13—H13	119.3	C27—C28—H28	120.0
C14—C13—H13	119.3	C23—C28—H28	120.0
			
O2—C1—C2—C3	−6.4 (2)	C15—C16—C17—C18	1.1 (2)
O1—C1—C2—C3	172.81 (15)	C16—C17—C18—C19	−0.3 (2)
O2—C1—C2—C7	174.49 (15)	C17—C18—C19—C20	−0.7 (2)
O1—C1—C2—C7	−6.2 (2)	C18—C19—C20—C15	1.0 (2)
C7—C2—C3—C4	2.3 (3)	C18—C19—C20—C21	−178.48 (15)
C1—C2—C3—C4	−176.81 (16)	C16—C15—C20—C19	−0.2 (2)
C2—C3—C4—C5	−1.0 (3)	C14—C15—C20—C19	−179.56 (14)
C3—C4—C5—C6	−1.2 (3)	C16—C15—C20—C21	179.29 (13)
C4—C5—C6—C7	2.0 (3)	C14—C15—C20—C21	−0.1 (2)
C5—C6—C7—C2	−0.7 (2)	N1—C8—C21—N2	−0.15 (16)
C3—C2—C7—C6	−1.4 (2)	C9—C8—C21—N2	179.75 (13)
C1—C2—C7—C6	177.64 (14)	N1—C8—C21—C20	178.83 (13)
C22—N1—C8—C21	−0.49 (16)	C9—C8—C21—C20	−1.3 (2)
C22—N1—C8—C9	179.62 (15)	C22—N2—C21—C8	0.74 (16)
C21—C8—C9—C10	−178.91 (14)	C22—N2—C21—C20	−178.15 (15)
N1—C8—C9—C10	1.0 (2)	C19—C20—C21—C8	−179.40 (15)
C21—C8—C9—C14	0.3 (2)	C15—C20—C21—C8	1.1 (2)
N1—C8—C9—C14	−179.82 (14)	C19—C20—C21—N2	−0.7 (2)
C14—C9—C10—C11	−1.0 (2)	C15—C20—C21—N2	179.84 (14)
C8—C9—C10—C11	178.22 (14)	C8—N1—C22—N2	0.96 (16)
C9—C10—C11—C12	0.3 (2)	C8—N1—C22—C23	−176.97 (14)
C10—C11—C12—C13	0.6 (2)	C21—N2—C22—N1	−1.05 (16)
C11—C12—C13—C14	−0.8 (2)	C21—N2—C22—C23	176.90 (14)
C12—C13—C14—C9	0.2 (2)	N1—C22—C23—C24	175.89 (14)
C12—C13—C14—C15	−179.05 (15)	N2—C22—C23—C24	−1.7 (2)
C10—C9—C14—C13	0.7 (2)	N1—C22—C23—C28	−3.0 (2)
C8—C9—C14—C13	−178.51 (13)	N2—C22—C23—C28	179.44 (14)
C10—C9—C14—C15	179.95 (14)	C28—C23—C24—C25	−1.2 (2)
C8—C9—C14—C15	0.7 (2)	C22—C23—C24—C25	179.97 (14)
C13—C14—C15—C16	−1.0 (2)	C23—C24—C25—C26	−0.1 (2)
C9—C14—C15—C16	179.84 (14)	C24—C25—C26—C27	1.0 (2)
C13—C14—C15—C20	178.35 (14)	C25—C26—C27—C28	−0.6 (2)
C9—C14—C15—C20	−0.8 (2)	C26—C27—C28—C23	−0.7 (2)
C20—C15—C16—C17	−0.8 (2)	C24—C23—C28—C27	1.5 (2)
C14—C15—C16—C17	178.53 (15)	C22—C23—C28—C27	−179.60 (14)

### Hydrogen-bond geometry (Å, º)

Cg4 is the centroid of the C15–C20 benzene ring.

**Table:**

*D*—H···*A*	*D*—H	H···*A*	*D*···*A*	*D*—H···*A*
N1—H1···O1	0.86	1.77	2.6159 (17)	168
N2—H2···O2^i^	0.86	1.83	2.6523 (16)	158
C7—H7···*Cg*4^ii^	0.93	2.79	3.585 (2)	145
C10—H10···O1	0.93	2.40	3.265 (2)	155
C19—H19···O2^i^	0.93	2.54	3.372 (2)	150
C24—H24···O2^i^	0.93	2.50	3.343 (2)	152
C28—H28···O1	0.93	2.48	3.365 (2)	159

Symmetry codes: (i) −*x*+1, −*y*+1, −*z*+1; (ii) −*x*, −*y*+1, −*z*+1.
